# Cost of diabetes and its complications: results from a STEPS survey in Punjab, India

**DOI:** 10.1186/s41256-023-00293-3

**Published:** 2023-04-07

**Authors:** Pooja Kansra, Sumit Oberoi

**Affiliations:** 1 Department Head of Economics, Mittal School of Business, Lovely Professional University, Punjab, India; 2 Symbiosis School of Economics, Symbiosis International University, Pune, India

**Keywords:** Diabetes, Costs, Complications, Healthcare, Economic burden, India

## Abstract

**Background:**

Diabetes mellitus is an obtrusive universal health emergency in developed and developing countries, including India. With the exponential rise of epidemiological conditions, the costs of treating and managing diabetes are on an upsurge. This study aimed to estimate the cost of diabetes and determine the determinants of the total cost among diabetic patients.

**Methods:**

This cross-sectional study was executed in the northern state of Punjab, India. It involves the multi-stage area sampling technique and data was collected through a self-structured questionnaire adapted following the “*WHO STEPS Surveillance*” manual. Mann–Whitney U and Kruskal–Wallis tests were performed to compare the cost differences in socio-demographic variables. Lastly, multiple linear regression was conducted to determine and evaluate the association of the dependent variable with numerous influential determinants.

**Results:**

The urban respondents' average direct and indirect costs are higher than rural respondents. Age manifests very eccentric results; the highest mean direct outpatient care expenditure of ₹52,104 was incurred by the respondents below 20 years of age. Gender, complications, income, history of diabetes and work status were statistically significant determinants of the total cost. Study reports a rapid increase in the median annual direct and indirect cost from ₹15,460 and ₹3572 in 1999 to ₹34,100 and ₹4200 in 2021.

**Conclusions:**

The present study highlights that the economic jeopardy of diabetes can be managed by educating people about diabetes and its associated risk factors. The economic burden of diabetes could be restrained by formulating new health policies and promoting the use of generic medicines. The result of the study directs that expenditure on outpatient care is to be reimbursed under the ‘*Ayushman Bharat-Sarbat Sehat Bima Yojana’*.

**Supplementary Information:**

The online version contains supplementary material available at 10.1186/s41256-023-00293-3.

## Background

The aetiological mechanisms of determining diabetes mellitus are multitudinous and multi-factorial, with biological, physical, behavioural and socio-economical determinants as imperative ones [1]. Being a metabolic syndrome, diabetes accounts for prolonged and multifarious repercussions such as the progression of microvascular and macrovascular complications, loss of quality of life, health deterioration and increasing economic burden [2]. According to Kansra and Oberoi [3], “*Diabetes affects individuals at the very onset of their productive age, reduces the life expectancy of people and financially impoverishes households*”.

The global assessment of adults living with diabetes was 463 million in 2019 (20–79 years), which is expected to be 700 million by the year 2045 [4]. Diabetes alone engendered healthcare expenditure of US$760 billion in 2019 and 79% of diagnosed individuals are natives of low and middle-income economies. The healthcare system of such economies is not endowed to handle the escalating menace of diabetes [5, 6]. A mammoth amount of $294.5 billion is alone spent by the United States of America in a year on patients diagnosed with diabetes mellitus and associated complications, which is the largest in the world, followed by the Republic of China [7]. It was observed that 8.4% of the total health spending in the Southeast Asian region is only spent on treatment and medicare for diabetes. India is the epicentre of diabetes and constitutes 87.8% of diabetic patients in the Southeast Asian region [2]. Contemplating the facts, epidemiological alteration in diabetes mellitus has an enormous economic burden on India. Healthcare expenditure at the country level on diabetes after revising purchasing power in India was US$31 billion in 2017, thereby pushing India to fourth position trailed by the United States of America, the Republic of China and Germany.

With the exponential rise of epidemiological conditions, the cost of treating and managing diabetes is on the upsurge [8]. Understanding the provision, utilization of healthcare services and expenditure is difficult, especially when the healthcare sector is diverse. The availability of cost data is scant for developing economies, including India [9]. Studies to estimate costs of diabetes and expenditure on outpatient/inpatient care comparison by socio-demographic characteristics and determining cost predictors are limited. Additionally, most prior articles assessing cost-of-diabetes (COD) used secondary data acquired from ‘*hospital databases*’ or ‘*national health surveys’*, which possess evident restraints regarding reliability [10, 11]. Therefore, this study aims to estimate the average annual costs of diabetes and determine the determinants of the total cost, where the cost data includes both outpatient and inpatient care. The findings of this study will provide the latest evidence on the economic burden incurred by the diabetics with and without complications, which will be a helpful aid in the planning of health care needs and resource allocation.

## Methods

### Study design

This cross-sectional study was executed in the northern state of Punjab, India. The survey employed a “*multistage area sampling*” technique by using population census data of statistical abstract of Punjab as the sample frame.

The sample size was assessed based on prior approximations of risk factors prevalence, a confidence interval of 95% and the margin of error of 5% was used as per the “*WHO STEPSwise Surveillance*” manual approach [12]. The calculated sample of 384 participants was amended for design effect (1.5). Further, by multiplying the value of the design effect (1.5), a sample size of 576 respondents was achieved which was passable to fend for state-wide results by age, gender and location (urban/rural). Finally, assuming a response rate to be 80%, the sample size was raised to 720 households.

$${\text{n}} = {\text{Z}}^{{2}} *\left( {\text{p}} \right)*\left( {\text{q}} \right)/{\text{e}}^{{2}}$$

The present study involves the multi-stage area sampling technique. The first phase of the sampling technique involves the geographical clustering of Punjab in 3 regions namely Majha, Doaba and Malwa. In the second phase, 50% of the districts were selected from each geographical cluster based on high and low per-capita income [3]. Finally, from each selected district both rural and urban areas were considered. A three-step sampling design was applied in rural areas. In the primary stage, one development block was selected based on the highest number of primary healthcare centres. In the second stage, two villages were selected from the development block nearer to PHCs. Finally, in the third stage respondents were randomly selected using the Kish table method [13]. A similar three-step approach was followed in an urban area. A city was selected based on the highest number of public/private hospitals.

Further, wards were selected by employing the probability proportional to size (PPS) method and one enumeration block (EB) was selected from each ward. Finally, respondents were randomly selected by using the Kish table method. Sixty primary sampling units (PSU) were observed, 20 villages and 40 enumeration blocks from the urban locality. From each selected PSU, 12 secondary sample units (SSUs) were selected [12].

### Variables and model specification

The cost of diabetes mellitus was estimated from the patient’s perspective, seeing direct and indirect costs as the substantial cost components. Direct costs can be defined as the costs related to the use of resources as a direct result of the diagnosis, treatment and healthcare procedures [2, 14]. Total direct cost is calculated by putting together the direct medical and direct non-medical costs. Direct medical costs comprise physician’s consultancy fee, diagnostic expenditure, medical spending on prescribed drugs and supplies. At the same time, direct non-medical cost incorporates hospitalization cost, transportation cost for visiting healthcare facility and expenditure on food and other material [15].

Indirect costs relate to the loss of productive working time both for the patient and the healthy household members who have to care for the patient [2, 15, 27]. The productive wage loss to attend outpatient and inpatient visits during hospitalization was recorded based on the responses provided by the patient and accompanying person. Therefore, the human capital approach has been applied in the present study [16, 17] and it's observed as the most reliable estimate. Indirect cost by human capital approach in this study is based on the wages loss due to absenteeism or the earnings lost by the patient/accompanying person because of illness.

In exponential and power regression four types of logarithmic transformations of variables is possible:

**Table:** 

Y	X	
	X	Log X
Y	Linear model	Linear-log model
	Y_i_ = α + β_1_X_1_ + β_2_X_2_ + ⋯ β_i_X_i_ + ε_i_	Y_i_ = α + β_1_logX_1_ + β_2_X_2_ + ⋯ β_i_X_i_ + ε_i_
Log Y	Log-linear model	Log–log model
	logY_i_ = α + β_1_X_1_ + β_2_X_2_ + ⋯ β_i_X_i_ + ε_i_	logY_i_ = α + β_1_ logX_1_ + β_2_X_2_ + ⋯ β_i_X_i_ + ε_i_

Log-linear regression model is applied for this study:

1 $$\begin{aligned} \log {\text{Y}}_{{\text{i}}} &= \alpha +\upbeta _{1} {\text{X}}_{1} +\upbeta _{2} {\text{X}}_{2} +\upbeta _{3} {\text{X}}_{3} +\upbeta _{4} {\text{X}}_{4}\\ &\quad +\upbeta _{5} {\text{X}}_{5} +\upbeta _{6} {\text{X}}_{6} +\upbeta _{7} {\text{X}}_{7} +\upbeta _{8} {\text{X}}_{8} +\upvarepsilon _{{\text{i}}}\end{aligned}$$

where log Y_i_ = total cost; X_1_ = gender, X_2_ = complications, X_3_ = marital status, X4 = family type, X5 = history of diabetes, X_6_ = income, X_7_ = education, X_8_ = work status and ε_i_ = error term.

### Questionnaire development

The primary data was collected through a self-structured questionnaire adapted using the “*WHO STEPSwise Surveillance*” manual [12]. A culturally adapted and pre-tested version of the WHO STEP Surveillance questionnaire in English was used after marginal modifications. A four-stage procedure for developing the study questionnaire was followed (1) designing and composition of the instrument (2) modification of items (3) testing of psychometric traits (4) reliability test. A detailed procedure for developing and adapting the study questionnaire can be found in a related research article [6]. The final draft of the questionnaire was corrected based on the feedback given by a diabetologist and a panel of five academicians. Thus, the final questionnaire includes a total of 27 questions distributed under three different sections. Section 1 highlights household identification and basic characteristics (20 questions). Diabetes complications and its types was discussed in section 2 (3 questions). Lastly, data on the cost of diabetes incurred during outpatient and inpatient care was observed in section 3 (4 questions) (Additional file 2: Appendix 1).

### Data collection and processing

The survey for the present study was executed from the first week of October 2021 till the third week of November 2021. Since this study is part of a PhD dissertation, the authors have themselves collected the responses. For the survey, a cluster of 50–70 households was identified randomly in an urban area, and the respondents of age 21–60 years were inquired randomly. Similarly, a cluster of 20–30 households was identified in a rural area, and respondents aged between 21 and 60 years were inquired about diabetes and its associated costs. Altogether, 720 responses were collected from 20 villages and 40 enumeration blocks of the ten districts of Punjab. The collected data was further entered into an excel spreadsheet for data documentation (Additional file 1: Table S1). In the very first spreadsheet, data was documented in predefined categories such as region, gender, age, history of diabetes and so on. The documented data was cleaned, coded and analysed using the statistical package for the social sciences (SPSS) version 23.

### Statistical analysis

Descriptive analyses were performed to highlight study respondents based on their social and demographic characteristics. Mean, median (range) and proportions for cost variables were reported in the study. Furthermore, 25th and 75th percentile was also estimated for costs of diabetes by components of direct and indirect cost. Data normality was checked by observing the Shapiro-Wilks test of normality. Since the cost data was positively or rightly skewed and *p* < 0.05, data was not normally distributed. Therefore, non-parametric tests viz*.* Mann–Whitney U and Kruskal–Wallis tests were performed to compare the cost differences in socio-demographic variables between the groups. Thus, a statistically significant level of difference was observed at *p* < 0.05. Multiple linear regression (MLR) was conducted to determine and evaluate the association of the dependent variable with numerous influential factors. In the multiple linear regression (MLR) model, the total cost was observed as the dependent variable and incorporated both direct and indirect cost components. Total cost was log10 transformed (TotalCost_Log) and regressed with independent variables. Categorical variables as independent variables (I.Vs) were converted into dummy variables.

## Results

### Characteristics of the study respondents

Table 1 of the study outlines the demographic and socio-economic profile of the respondents. Out of 720 respondents, a large proportion of study respondents were males and 38% of respondents were females. A majority of study respondents were from the age group 41–60 years and were having secondary education followed by primary, graduation, post-graduation, illiterates and others. It was found that large proportion of respondents were businessman and only 4% respondents were students. The results revealed that 54% respondents had history of diabetes in their family and 71% of the study respondents don’t know about the type of diabetes they were diagnosed with. Whereas, 23% respondents acknowledge of being diagnosed with Type-2 diabetes. Table 1 shows the profile of diabetic respondents based on complications in Punjab. It was found that 52% respondents were diagnosed with any of the complication due to diabetes. Moreover, it was observed that 61% of the respondents were diagnosed with microvascular complications and 39% with macrovascular complications. The result of the analysis shows that out of 201 respondents with macrovascular complications, 48% of the respondents were diagnosed with CAD followed by PVC (40%), hypoglycaemia (9%) and TIA (2%). However, under microvascular complications it was found that 55% of the respondents suffer retinopathy followed by periodontitis (21%), vasculopathy (12%), nephropathy (06%) and neuropathy/foot ulcer (3%).

**Table 1:** Socio-demographic profile of the study respondents, Punjab

Characteristics	(N = 720) (%)
*Gender*	
Male	445 (62)
Female	275 (38)
*Residence*	
Urban	480 (67)
Rural	240 (33)
*Marital status*	
Single	96 (13)
Married	624 (87)
*Age group*	
Upto 20 years	19 (03)
21–40 years	93 (13)
41–60 years	360 (50)
60 years and Above	248 (34)
*Work status*	
Salaried	81 (11)
Business	274 (38)
Student	32 (04)
Homemaker	213 (30)
Retired	61 (09)
Other	59 (08)
*Education level*	
Illiterate	41 (06)
Primary	218 (30)
Secondary	223 (31)
Graduate	189 (26)
Post-graduate	44 (06)
Other	05 (01)
*Monthly income*	
Less than ₹15,000	107 (15)
₹ 15,000–₹ 30,000	232 (32)
₹ 30,000–₹ 45,000	169 (24)
₹ 45,000–₹ 60,000	104 (14)
₹ 60,000 and above	108 (15)
*Family type*	
Nuclear	342 (47)
Joint	378 (53)
*History of diabetes in family*	
Yes	384 (54)
No	336 (46)
Diabetic in family*	
Mother	144 (30)
Father	113 (23)
Both parents	69 (14)
Sibling	133 (28)
Children	22 (05)
*Type of diabetes*	
Type-1	46 (06)
Type-2	159 (23)
Gestational diabetes	02 (00)
Don’t know	513 (71)
*Complications*	
Yes	374 (52)
No	346 (48)
Macrovascular complications*	
Coronary artery disease (CAD)	97 (48)
Transient ischemic attack (TIA)	04 (02)
Hypoglycaemia	19 (09)
Peripheral vascular disease (PVC)	81 (41)
Microvascular complications*	
Foot ulcer	10 (03)
Periodontitis complication	77 (21)
Retinopathy	205 (55)
Neuropathy	10 (03)
Nephropathy	24 (06)
Vasculopathy	45 (12)

Source: Authors calculation based on primary data

*Multiple responses possible

### Annual cost of diabetes for inpatient and outpatient care

Details of annual inpatient and outpatient care expenditure of diabetes by socio-demographic characteristic is highlighted in Table 2. The average direct and indirect costs are higher among the urban respondents, which is significantly different from the costs incurred by rural respondents with *p* < 0.05. The mean direct and indirect cost of female respondents were higher in comparison to male respondents under outpatient care. Age manifests eccentric results, respondents below 20 years of age incurred the highest mean direct outpatient care expenditure of ₹52,104, but respondents of age 21–40 years experienced the highest mean indirect cost. Under inpatient care section respondents of age 60 years and above incurred enormous direct (₹41,482) and indirect costs (₹4818).

**Table 2:** Annual direct and indirect cost by socio-demographic characteristics of the patients utilizing outpatient and inpatient care

Socio-demographic variable	Outpatient care				Inpatient care			
	Direct cost		Indirect cost		Direct cost		Indirect cost	
	₹Mean (median)	*p*-Value	Mean (median)	*p-*Value	₹Mean (median)	*p-*Value	Mean (median)	*p-*Value
*Region*								
Urban	40,752 (30,000)*	0.000	9030 (9000)*	0.011	40,643* (24,000)	0.002	4618* (3000)	0.035
Rural	24,252 (18,000)		7884 (7200)		23,568 (10,000)		3160 (2400)	
*Gender*								
Male	34,080 (30,000)	0.009	9600 (8400)	0.811	45,045 (23,000)	0.117	4980* (3200)	0.007
Female	37,140 (30,000)*		10,236 (8400)		27,269 (20,000)		3511 (2500)	
*Age*								
Below 20 years	52,104 (30,000)**	0.009	10,704 (9600)	0.234	17,750 (16,500)	0.001	2900 (2900)	0.301
21–40 years	37,368 (36,000)		11,124 (8400)		11,119 (6000)		2487 (2000)	
41–60 years	34,704 (30,000)		10,176 (8400)		35,169 (21,600)		4133 (3000)	
60 years and Above	33,960 (26,400)		8280 (7200)		41,482** (24,000)		4818 (3000)	
*Marital status*								
Single	35,604 (33,600)	0.359	9672 (8400)	0.221	35,946 (22,000)	0.601	3937 (3000)	0.625
Married	35,196 (30,000)		9852 (8400)		35,972 (20,000)		4218 (2900)	
*Education*								
Illiterate	22,116 (18,000)	0.000	6516 (5400)	0.000	33,200 (22,000)	0.336	4170 (1200)	0.056
Primary	26,784 (22,500)		8004 (7200)		27,518 (18,500)		3179 (2400)	
Secondary	36,288 (30,000)		8664 (7200)		39,324 (23,500)		4706 (3000)	
Graduation	41,940 (36,000)		11,448 (9600)		35,229 (20,000)		4845 (3200)	
Post-graduation	50,496 (49,600)		15,912 (14,400)		59,364 (30,000)		3778 (4000)	
Other’s	52,320** (51,400)		16,800** (14,400)		64,000 (64,000)		1400 (1200)	
*Work status*								
Salaried	41,352 (39,600)	0.000	11,788 (8400)	0.001	21,581 (14,000)	0.008	3747 (3200)	0.000
Business	35,508 (30,000)		8556 (8400)		53,808** (32,000)		5851** (4800)	
Student	47,664** (43,956)		12,156** (9600)		16,033 (16,500)		2591 (3000)	
Homemaker	33,768 (27,000)		9912 (8400)		27,197 (18,500)		3431 (2400)	
Retired	39,732 (32,400)		11,172 (8400)		32,236 (22,000)		3873 (3350)	
Other’s	19,692 (15,600)		6204 (6000)		26,225 (29,500)		2590 (2500)	
*Income*								
Less than ₹15,000	17,964 (14,400)	0.000	6036 (6000)	0.000	17,467 (15,000)	0.000	1918 (2000)	0.000
₹15,000–₹30,000	28,188 (25,800)		8244 (7200)		28,799 (17,000)		3578 (2500)	
₹30,000–₹45,000	37,368 (34,800)		9420 (8400)		30,732 (20,000)		4132 (3200)	
₹45,000–₹60,000	43,752 (38,400)		10,260 (9600)		53,633 (32,600)		5196 (4000)	
₹60,000 and above	55,788** (51,600)		16,080** (12,000)		57,394** (35,000)		7162** (5250)	
*Family type*								
Nuclear	32,784 (28,800)	0.065	10,116 (8400)	0.489	34,512 (20,000)	0.628	4288 (3000)	0.932
Joint	37,452 (30,000)		9552 (8400)		37,254 (23,000)		4267 (3000)	
*History of diabetes*								
Yes	36,936* (32,700)	0.013	9948 (8400)	0.804	37,104* (24,000)	0.024	3942 (3000)	0.336
NO	33,336 (26,400)		9636 (8400)		34,761 (18,500)		4665 (2600)	
*Complications*								
Yes	41,940* (36,000)	0.000	10,728* (9600)	0.004	41,875* (25,000)	0.000	4819* (3000)	0.001
NO	28,032 (24,000)		8460 (7200)		15,162 (11,000)		2525 (2400)	
*Household size*								
Upto 3 Members	34,896 (32,400)	0.012	9576 (8400)	0.851	27,169 (18,500)	0.028	3654 (2000)	0.508
4–6 Members	33,156 (29,400)		9804 (9600)		30,661 (18,500)		3964 (3000)	
6 Members and Above	41,592** (30,000)		9948 (9600)		52,219** (31,400)		5302 (3000)	

Source: Authors calculation established on primary data

Direct cost includes consultation fees, medicine expenditure, hospitalization fees, diagnostic expenditure, transportation costs, food and other materials and others (specify)

Indirect cost includes the patient’s wage loss and the wage loss of the accompanying person

^*^Mann Whitney U test and **Kruskal Wallis test done for group comparison; *p*-value was considered significant at *p* < 0.05

With the increase in level of education both direct and indirect costs under outpatient care increased constantly, thus revealing statistically significant difference (*p* = 0.000). Respondents with high-income levels spend more on outpatient and inpatient care as compared to low-income level individuals. History of diabetes in the family plays an imperative role in determining the cost of diabetes. Respondents with history of diabetes spent more on direct outpatient (₹36,936) and inpatient care (₹37,104) as compared to respondents with no history of diabetes (*p* = 0.013; *p* = 0.024). Lastly, respondents with complications spent significantly higher direct and indirect annual outpatient (₹41,940, *p* = 0.000; ₹10,728, *p* = 0.004) and inpatient care (₹41,875, *p* = 0.000; ₹4819, *p* = 0.001), which is statistically significant at *p* < 0.05.

### Annual cost of diabetes by component of direct and indirect cost in Punjab

Table 3 estimates the direct and indirect annual costs of diabetes. The mean total cost of diabetes was ₹49,037 of which the total direct cost was 93% and the total indirect cost was 7%. The 25th percentile of the total cost of diabetes highlights that 75% of the total cost is as large or larger than ₹60,600 and 25% of the total cost is as small or smaller than ₹19,200. Expenditure on medicine was the highest cost component of diabetes and accounts for 64% under the direct cost and 60% under the total cost of diabetes.

**Table 3:** Annual cost of diabetes by component of direct and indirect cost (₹) in Punjab

Cost component	Mean	Median	25th Percentile	75th Percentile	% Of total direct and indirect cost	Total cost of diabetes	% Of total cost of diabetes
1. Annual overall cost-of-diabetes (COD)							
*1.1. Direct cost*							
Consultation fee	3992	3175	2400	4800	05	1,772,480	05
Medical expenditure	29,391	24,050	14,200	38,500	64	21,161,420	60
Cost of hospitalization	5785	3500	1575	7775	04	1,168,550	03
Diagnostic expenditure	9316	6600	3600	11,750	13	4,453,210	13
Transportation cost	1622	1000	600	1537	01	366,670	01
Food and other material	2632	2000	1000	3500	02	492,100	02
Other's	33,427	20,000	10,000	45,000	11	3,409,600	09
Total direct cost	₹45,915	₹34,100	₹18,000	₹56,300	100	₹32,824,030	93
*1.2. Indirect cost*							
Patient’s wage loss	5402	4000	2150	7200	52	1,145,200	04
Wage loss of accompanying person	4946	3100	2400	5800	48	1,122,800	03
Total indirect cost	₹5884	₹4200	₹2400	₹7200	100	₹2,268,000	07
Total cost	₹49,037	₹36,000	₹19,200	₹60,600		₹35,092,030	
2. Annual cost of diabetes for outpatient care (n = 720)							
*2.1. Direct cost*							
Consultation fee	3768	3600	2400	4800	6	1,466,040	5
Medical expenditure	27,797	24,000	12,000	36,000	80	20,013,720	75
Diagnostic expenditure	8003	6000	3600	9600	13	3,377,400	12
Transportation cost	1854	1200	600	1800	1	313,320	1
Total direct cost	₹35,253	₹30,000	₹15,840	₹48,000	100	₹25,170,480	95
*2.2. Indirect cost*							
Patient’s wage loss	4668	4200	2400	4800	54	751,500	03
Wage loss of accompanying person	5110	4800	3000	6000	46	643,800	02
Total indirect cost	₹4909	₹4200	₹2400	₹6000	100	₹1,395,300	05
Total cost	₹37,169	₹30,600	₹16,800	₹48,000		₹26,565,780	
3. Cost of diabetes in past 365 days for inpatient care (n = 204)							
*3.1. Direct cost*							
Consultation fee	1751	1200	500	2500	4	306,440	4
Medical expenditure	5516	4000	2500	7200	16	1,097,700	14
Cost of hospitalization	5784	3500	1550	7850	17	1,162,550	15
Diagnostic expenditure	5478	4000	2000	7000	15	1,040,810	13
Transportation cost	684	375	200	700	1	53,350	1
Food and other material	2632	2000	1000	3500	7	492,100	6
Other's	30,963	18,000	8000	43,250	41	2,879,600	36
Total direct cost	₹35,970	₹20,000	₹9000	₹40,000	100	₹7,032,550	89
*3.2. Indirect cost*							
Patient’s wage loss	3172	3000	1200	4150	44	383,700	5
Wage loss of accompanying person	3837	2500	1500	3200	56	479,000	6
Total indirect cost	₹4277	₹3000	₹1800	₹5000	100	₹862,700	11
Total cost	₹41,681	₹24,500	₹11,550	₹47,600		₹7,895,250	

Source: Authors calculations established on primary data

Cost of hospitalization includes room charges during hospital stay and ICU charges

Other’s direct cost includes cost incurred on surgeries because of diabetes or associated micro and macro-vascular complications

Table 3 also revealed the mean total cost of outpatient care is ₹37,169, out of which total direct cost accounts for 95% and indirect cost for outpatient care accounts for the rest 5% of the annual cost. Expenditure on medicines was reported as the highest cost component (80%) of direct cost under outpatient care followed by diagnostic expenditure, consultation fees and transportation costs. Furthermore, it was found that wage loss incurred by diabetic respondents was 54% under indirect costs under outpatient care.

The analysis revealed that out of 720 respondents, only 204 respondents utilized inpatient care. It was found that others (surgery) was the highest direct cost component for inpatient care. The 25th percentile of the total inpatient expenditure of diabetes exhibits that 75% of the total costs are as large or larger than ₹47,600 and 25% of the total cost on inpatient care are as small or smaller than ₹11,550. The mean indirect cost was ₹4277 and reports only an 11% share of the total cost under inpatient care. The mean wage loss of the accompanying person was ₹3837 as compared to the wage loss of diabetic respondents (₹3172).

### Comparison of inpatient care expenditure among diabetics with and without complications

Table 4, reports the details of inpatient care expenditure for treating diabetics with and without complications in Punjab. Respondents were divided into four different categories such as respondents without complications (group-1), respondents with microvascular complications (group-2), macrovascular complications (group-3) and lastly, respondents with both micro and macrovascular complications (group-4).

**Table 4:** Details of inpatient care expenditure for treating diabetics with and without complications in Punjab

Cost variables	Group 1 (without complications)		Group 2 (with micro-vascular complications)		Group 3 (with macro-vascular complications)		Group 4 (with both complications)	
	Mean	Median (range)	Mean	Median (range)	Mean	Median (range)	Mean	Median (range)
Consultation fee (₹)	1365	700 (5600–100)	1713	1200 (8000–20)	1949	1500 (8000–200)	1650	1200 (5000–20)
Expenditure on medicines (₹)	4058	3400 (12,000–500)	5694	4000 (25,000–500)	6364	4800 (35,000–800)	5603	3650 (18,000–1000)
Hospitalization fees	5079	2250 (24,000–750)	5077	3500 (25,000–200)	6518	4900 (25,000–200)	4802	3000 (18,000–200)
Diagnostic expenditure (₹)	3561	2500 (12,600–250)	5495	4000 (42,000–250)	6547	5000 (24,000–250)	5345	3750 (18,000–250)
Transportation (₹)	322	350 (700–100)	906	500 (12,000–50)	533	4800 (4800–50)	452	275 (1000–50)
Food and other material	1516	1000 (5000–300)	2659	2000 (8000–200)	3032	2500 (9000–300)	2513	2000 (8000–300)
Other’s (Surgeries, etc.)	16,447	15,000 (35,500–200)	31,578	20,000 (160,000–200)	41,099	25,500 (160,000–200)	32,842	30,000 (155,000–200)
Total direct cost (₹)	20,767	14,000 (70,000–2500)	36,412	21,800 (234,000–3000)	48,001	28,000 (250,000–3000)	36,748	24,000 (200,000–3000)
Wage loss incurred by diabetic patients (₹)	2416	2000 (4500–400)	3411	2400 (18,000–400)	4587	3000 (18,000–500)	3251	1800 (18,000–500)
Wage loss incurred by accompanying person (₹)	2429	2000 (7400–400)	3496	2800 (20,000–300)	3127	2500 (20,000–300)	3260	2000 (20,000–300)
Total indirect cost (₹)	2525	2400 (7400–400)	4226	3000 (20,000–500)	5291	3150 (24,000–700)	4509	2750 (20,000–800)

Source: Authors calculation established on primary data

Group-1: No Complications (n = 48)

Group-2: Microvascular complications (foot ulcer, retinopathy, neuropathy, nephropathy, periodontitis, vasculopathy) (n = 118)

Group-3: Macrovascular complications (coronary artery disease, transient ischemic attack, hypoglycaemia, peripheral vascular disease) (n = 89)

Group-4: Microvascular and Macrovascular Complications (n = 52)

The mean total direct cost of diabetes was highest under macrovascular complications (group-3) followed by group-4 (both complications), group-2 (microvascular complications) and group-1 (without complications). It was found that respondents with microvascular and macrovascular complications witnessed other, diagnostic expenditures and expenditure on medicines as the top three cost components of direct cost. The mean total indirect cost of respondents with macrovascular complications (₹5291) was highest as compared to the group with both complications (₹4509), microvascular complications (₹4226) and without complications group (₹2525). The results of the analysis revealed that the mean wage loss incurred by respondents with macrovascular complications (group-1) was highest ₹4587 followed by microvascular complications (₹3411), both complications group (₹3251) and without complications (₹2416) group respondents. However, the average wage loss incurred by the accompanying person was highest amongst the respondents under group-2 (microvascular complications).

### Determinants of the total costs of diabetic patients

Multiple linear regression (MLR) analysis techniques was performed for determining the total cost (Table 5). A statistically significant regression equation with an r^2^ value of 0.696 highlights a 69% possibility of attaining the correct prediction of the total cost with independent variables. Durbin-Watson’s statistic value of 1.856 highlights the residuals are independent or uncorrelated. Similarly, collinearity statistic values under VIF and Tolerance section are well below 10 and above 0.2, thereby highlighting no multi-collinearity amongst the predictors or independent variables (IVs). Cook’s Distance statistic (Minimum = 0.000; Maximum = 0.059) lucidly explains that there exist no influential cases biasing the MLR model. Lastly, patients predict total cost (TotalCost_Log) is equal to 3.117 + 0.085 (Gender) + 0.159 (Complications) + 0.072 (History of Diabetes) + 0.176 (₹15,000–₹30,000) + 0.245 (₹30,000–₹45,000) + 0.311 (₹45,000–₹60,000) + 0.366 (₹60,000 and above) − 0.188 (Primary) − 0.203 (Secondary) + 0.022 (Graduation) + (Post-Graduation) + 0.119 (Salaried) + 0.198 (Student). The mentioned independent variables (IVs) were established to be statistically significant determinants of the total cost (Table 5).

**Table 5:** Multiple linear regression analysis for determinants of total cost of diabetic patients in Punjab

Model summary					
Model	R	R^2^	Adjusted R^2^	SE of the estimate	Durbin–Watson
1	0.774	0.696	0.672	0.24853	1.856

ANOVA					
	Sum of squares	d.f	Mean square	F	Sig
Regression	27.789	31	1.323	21.423	0.000
Residual	28.290	689	0.062		
Total	56.079	720			

Coefficients					
Variables	β	SE	*p*-Value	Collinearity statistic	
				Tolerance	VIF
Constant	3.117	0.085	0.000	–	–
Gender (male = 1, female = 0)	0.085	0.038	0.028	0.358	2.790
Complications (yes = 1, no = 0)	0.159	0.024	0.013	0.976	1.025
Marital status (married = 1, single = 0)	0.064	0.040	0.115	0.766	1.306
Family type (joint = 1, nuclear = 0)	0.037	0.027	0.176	0.683	1.463
History of diabetes (yes = 1; no = 0)	0.072	0.024	0.003	0.872	1.146
*Income*					
₹15,000–₹30,000	0.176	0.046	0.000	0.320	2.126
₹30,000–₹45,000	0.245	0.046	0.000	0.326	3.072
₹45,000–₹60,000	0.311	0.049	0.000	0.350	2.855
₹60,000 and above	0.366	0.050	0.000	0.300	3.333
*Education*					
Primary	− 0.188	0.044	0.000	0.391	2.560
Secondary	− 0.203	0.043	0.000	0.363	2.753
Graduation	0.022	0.041	0.030	0.349	1.866
Post-graduation	0.048	0.026	0.031	0.583	1.716
Other’s	0.157	0.072	0.331	0.869	1.150
*Work status*					
Salaried	0.119	0.055	0.032	0.302	3.315
Business	0.021	0.058	0.719	0.158	6.323
Student	0.198	0.078	0.011	0.468	2.137
Homemaker	0.039	0.069	0.574	0.130	7.688
Retired	0.082	0.070	0.242	0.376	2.660

Cooks distance statistic (minimum = 0.000; maximum = 0.059)

## Discussion

The economic menace of diabetes is a major global concern in recent decades, because of the chronicity and existence of multiple comorbidities. Though diabetes is seen as an economic and epidemiological threat in India, still there is a dearth of literature on adequate assessment of the cost of diabetes. Escalating the incidence and peril of diabetes is a foremost apprehension for India owing to rapid behavioural and socio-demographic modifications viz. dietary alteration, urbanization, obesity and sedentary lifestyle results in the incessant occurrence of diabetes in India. According to Rice [18], “*Cost epitomize the financial burden of illness on society and preterm mortality*”. Numerous policymakers, academicians and health planners consider cost estimation as a foremost criterion for decision making, determining urgency and considering health budgets.

Traces of cost-of-diabetes (COD) literature in India was first witnessed in a Bangalore-based study in 1999, the direct and indirect annual cost was reported to be ₹15,460 and ₹3572 [19]. Numerous population-based studies from 2000 to 2009 assessed the median yearly direct cost to be ₹9053, ranging from ₹7070 to ₹14,000 and the median yearly indirect cost was ₹4681, ranging from ₹2435 to ₹12,756 [20–24]. A swift augmentation in the COD was witnessed in a total of 18 studies published from 2010 to 2020, which estimated the median yearly direct cost to be more than twice accounting to be ₹21,082, ranging from ₹4282 to ₹76,779 and the median yearly indirect cost was ₹7443, ranging from ₹1198 to ₹30,670 [8]. The annual median direct and indirect overall cost-of-diabetes reported in the current study is ₹34,100 and ₹4200. The cost estimation results of the present study are akin to a recent population-based study conducted in Shillong, Meghalaya [25]. According to International Diabetic Federation [4], expenditure on diabetes in India at the per-person level was assessed to be ₹6900 (US$92), significantly less than reported by various available literature and also than the existing study. Therefore, it’s completely pertinent having regard to the fact that the South Asian population especially Indians got more affected by diabetes and its comorbidities a decade earlier, engendering to “quality-adjusted life-year” (QALY) and long treatment duration [26, 27].

The findings of the study reflect upon the rising cost of treatment associated with high-income levels, educational background, complications and history of diabetes. The study findings are analogues with the existing literature inferring that individuals with high per capita income and literacy rate are more vulnerable to diabetes because of sedentary lifestyle and low physical exercise [28, 29]. The results of the study report that median annual cost-of-diabetes under outpatient care is significantly identical to overall cost-of-diabetes, highlighting medical spending and diagnostic expenditure as biggest drivers of treatment. Studies at national and international level, embracing review article by Yesudian et al. [30] and Oberoi and Kansra [8], shows medication expenditure as an important cost component of overall and outpatient treatment, but cost components under inpatient care are divergent. Under inpatient median annual cost-of-diabetes “Other’s” ₹18,000 was the leading cost component, followed by medical/diagnostic expenditure and median hospitalization cost as the major cost component.

Diabetes is accompanied by severe and prolonged comorbidities, which is a foremost cause of illness, hospitalization and early fatality [31–33]. Complications associated with diabetes are majorly categorized into microvascular and macrovascular illnesses [31, 34]. In the direction of conversing the outcomes of the present study, it’s worth citing that the total direct cost-of-diabetes for treating groups with complications is on an upsurge. The results of the study manifested that the median direct and indirect cost of managing and treating groups with macrovascular complications was highest in comparison to group-4, group-2 and group-1 respondents. With the incidence of a single macrovascular comorbid illness viz*.* CAD the mean and median direct cost of managing diabetes increased by more than twice in comparison to those patients without complications (Table 4). Diabetic with microvascular complications stands second in the highest direct cost ranking and holds first rank under “*Cost Incurred by Accompanying Person*” of total indirect cost. As expected, the respondents under group-1 (without complications) had low cost-of-diabetes for all cost components, patients under group-1 were comparatively younger and were recently diagnosed with diabetes. The results of the present study defend the well-established fact that comorbidities aggravate with prolongation of diabetes mellitus.

The multiple linear regression (MLR) model for identifying determinants of the total cost displayed an explicit role of complications in the current research work, thus suggesting hasty diagnosis, swift management and treatment of identified comorbidities. Misra et al. [27], highlighted South Asian population to be susceptible to early onset of diabetes and risk of developing associated complications and ultimately the increased treatment cost. Other significant determinants of total cost were diagnostic cost, region, high level of income and work status, which corroborates with the conclusions of available literature [2, 5, 15, 31].

There are few limitations to the present study that need to be addressed. Primarily, along with both direct and indirect costs, there exists intangible costs such as loss of quality life, pain, suffering, etc. which are not estimated in this study. Secondly, costs of illness were estimated using descriptive statistics that doesn’t provide evidence on the efficient use of resources, therefore, higher cost doesn’t highlight more advanced or better healthcare services. Lastly, the cost estimates for both direct and indirect costs were majorly based on patients recall method which could have respondents recall bias.

## Conclusions

The discussion accentuated upon a colossal economic encumbrance of diabetes mellitus in Punjab and cost variations were detailed in different segments. Under direct cost-of-diabetes it was witnessed that expenditure on medicines was regarded as a major cost component under outpatient care. The study also reported a rapid increase in the median annual direct and indirect cost from ₹15,460 and ₹3572 in 1999 to ₹34,100 and ₹4200 reported in the current study, which is approximately 2.5 times for direct cost and 1.2 times for indirect costs. A similar trend and pattern were also witnessed under the complications section. Individuals under group-3 (macro-vascular complications) account for twice the cost burden incurred by group-1 patients.

The economic haunting of the diabetes could be restrained by early identification and prevention of diabetes complications. To reduce costs of outpatient care reimbursement should be made under the ‘*Ayushman Bharat-Sarbat Sehat Bima* scheme and promoting the use of generic medicines. Since diabetes mellitus is a lifestyle syndrome, alterations in dietary habits, physical activity and behavioural modification could help in reducing the economic menace.

### Supplementary Information

**Additional file 1.** Table S1.

**Additional file 2.** Appendix 1.

## Data Availability

Available if requested.

## Abbreviations

CAD: Coronary artery disease
COD: Cost-of-diabetes
EB: Enumeration block
IVs: Independent variables
MLR: Multiple linear regression
PHCs: Primary Healthcare Centres
PPS: Probability proportional to size
PSU: Primary sampling unit
QALY: Quality-adjusted life-year
SSU: Secondary sampling unit
VIF: Variance inflation factor
WHO: World Health Organization
