# Correction: Cost of diabetes and its complications: results from a STEPS survey in Punjab, India

**DOI:** 10.1186/s41256-023-00322-1

**Published:** 2023-08-23

**Authors:** Pooja Kansra, Sumit Oberoi

**Affiliations:** 1 Department Head of Economics, Mittal School of Business, Lovely Professional University, Punjab, India; 2 Symbiosis School of Economics, Symbiosis International University, Pune, India

**Correction to: Global Health Research and Policy (2023) 8:11** 10.1186/s41256-023-00293-3

Following publication of the original article [1], the authors reported that the fifth sentence in the last paragraph of the Background section needed to be updated.

The original sentence was:

“Additionally, most prior articles assessing cost-of-diabetes (COD) used secondary data acquired from ‘*hospital databases*’ or ‘*national health surveys*’, which possess evident restraints regarding reliability [10–12].”

The updated sentence is:

“Additionally, most prior articles assessing cost-of-diabetes (COD) used secondary data acquired from ‘*hospital databases*’ or ‘*national health surveys*’, which possess evident restraints regarding reliability [10–11].”

The original article [1] has been corrected.
