# Supplementary material for: Cost of diabetes and its complications: results from a STEPS survey in Punjab, India
Source: Glob Health Res Policy. 2023 Apr 7;8:11. doi: 10.1186/s41256-023-00293-3 (PMC10080818; doi:10.1186/s41256-023-00293-3)
Supplement: Supplementary file 2 — Additional file 2. Appendix 1. [file 41256_2023_293_MOESM2_ESM.docx]

**Appendix 1**

**Section 1: Household Identification & Basic Characteristics**

| **S. No** | **Question** | **Response** |
| --- | --- | --- |
| 1. | Name of the Village/Ward |  |
| 2. | Household number |  |
| 3. | Name of the respondent |  |
| 4. | Mobile No. |  |
| 5. | Date of visit |  |
| 6. | Landmark for future identification |  |

| **S. No** | **Question** | **Response** | **Coding** |
| --- | --- | --- | --- |
| 1. | District | Amritsar = 1  Ferozepur = 2  Gurdaspur = 3  Jalandhar = 4  Ludhiana = 5  Mansa = 6  Rupnagar = 7  S.A.S = 8  S.B.S = 9  Shri Muktsar = 10 |  |
| 2. | Region | Urban =1  Rural = 2 |  |
| 3. | Gender | Male = 1  Female = 2 |  |
| 4. | Age | Upto 20 years = 1  21-40 years = 2  41-60 years = 3  Above 60 years = 4 |  |
| 5. | Marital Status | Single = 1  Married = 2 |  |
| 6. | Education Level | Illiterate = 1  Primary = 2  Secondary = 3  Graduation = 4  Post-Graduation = 5  Other (specify) = 6………………… |  |
| 7. | Present Work Status | Salaried = 1  Business = 2  Student = 3  Homemaker = 4  Retired = 5  Others(specify) = 6……………… |  |
| 8. | Monthly Household Income | Less than ar15,000 = 1  ₹15,000- ₹30,000 = 2  ₹30,000- ₹45,000 = 3  ₹ 45,000- ₹ 60,000 = 4  ₹ 60,000 and Above =5 |  |
| 9. | Family Type | Nuclear =1  Joint = 2 |  |
| 10. | Household Size | Up to 3 members = 1  4 to 6 members = 2  6 members and above = 3 |  |
| 11. | Average monthly household expenditure (in Rupees) | Food items  Clothing  Utilities (electricity, recharge, water bills, cable etc.)  Education  Rent  Recreation  Saving  Travelling  Miscellaneous |  |
|  |  |  |  |
|  |  |  |  |
|  |  |  |  |
|  |  |  |  |
|  |  |  |  |
|  |  |  |  |
|  |  |  |  |
| 12. | Is there any history of diabetes in your family? | Yes = 1  No = 2 |  |
| **If No, skip to section 3** | | | |
| 13. | If yes, who is diabetic in family? | Mother = 1  Father = 2  Both Parents = 3  Sibling = 4  Children=5 |  |
| 14. | If yes, please specify the type of diabetes they have? | Type-1 = 1  Type-2 = 2  Gestational = 3  I don’t know = 4 |  |

**Section 2: Complications of Diabetes Mellitus**

2.1. Do you know that diabetes can cause complications in other organs? Yes [ ] No [ ].

2.2. Do you suffer from any of the complication due to diabetes? Yes [ ] No [ ].

2.3. If yes, please specify the complications you have due to diabetes.

| **S. No** | **Type of Complications** | **(Multiple Response Possible)** |
| --- | --- | --- |
| **Macrovascular Complications** | | |
| 1. | Coronary Artery Disease (CAD) |  |
| 2. | Transient Ischemic Attack (TIA) |  |
| 3. | Stroke |  |
| 4. | Peripheral Vascular Disease (PVD) |  |
| **Microvascular Complications** | | |
| 1. | Foot Ulcer |  |
| 2. | Periodontitis Trending Complication |  |
| 3. | Retinopathy |  |
| 4. | Neuropathy |  |
| 5. | Nephropathy |  |
| 6. | Vasculopathy |  |

**Section 3: Details of Costs Incurred on Outpatient Care**

| **Costs** | **S. No.** | **Questions** | **Responses** |
| --- | --- | --- | --- |
| **3.1 Direct Cost** | 1 | Consultation fee |  |
|  | 2 | Expenditure on medicines |  |
|  | 3 | Diagnostic expenses |  |
|  | 4 | Transportation cost |  |
|  |  | **Total Direct Cost** |  |
| **3.2 Indirect Cost** | 1 | Did you take leave to seek treatment? | Yes [ ] No [ ] |
|  | **If No, skip to question 4** | | |
|  | 2 | If yes, how many man days did you loss? | Days…….. |
|  | 3 | How much money did you lose? | (₹)………… |
|  | 4 | Did the accompanying person took leave? | Yes [ ] No [ ] |
|  | **If No, skip to question 7** | | |
|  | 5 | If yes, how many man days did he/she loss? | Days……. |
|  | 6 | How much money did he/she loss? | (₹)………… |
|  | 7 | Other (specify)………… |  |
|  |  | **Total Indirect Cost** |  |

**Details of Cost Incurred for Inpatient Care**

| **Costs** | **S. No.** | **Questions** | **Responses** |
| --- | --- | --- | --- |
| **3.3 Direct Cost** | 1 | Consultation fee |  |
|  | 2 | Expenditure on medicines |  |
|  | 3 | Cost of Hospitalization |  |
|  | 4 | Diagnostic expenses |  |
|  | 5 | Expenditure incurred on ambulance |  |
|  | 6 | Food and other material |  |
|  | 7 | Other (specify)………. |  |
|  |  | **Total Direct Cost** |  |
| **3.4 Indirect Cost** | 1 | Did you take leave to seek treatment | Yes [ ] No [ ] |
|  | **If No, skip to question 4** | | |
|  | 2 | If yes, how many man days did you loss? | Days…….. |
|  | 3 | How much money did you lose? | (₹)………… |
|  | 4 | Did the accompanying person took leave? | Yes [ ] No [ ] |
|  | **If No, skip to question 7** | | |
|  | 5 | If yes, how many man days did he/she loss? | Days……. |
|  | 6 | How much money did he/she loss? | (₹)………… |
|  | 7 | Expenditure incurred on attending visitors | (₹)………… |
|  | 8 | Other (specify)………. |  |
|  |  | **Total Indirect Cost** |  |
